# Clinical and prognostic features of MMP-2 and VEGF in AEG patients

**DOI:** 10.1515/med-2021-0252

**Published:** 2021-05-14

**Authors:** Qing-Kang Zheng, Qing Yin, Nan Zhang, Zhi-Gang Sun

**Affiliations:** School of Clinical Medicine, Weifang Medical University, Weifang 261053, People’s Republic of China; Department of Medical Education, Jinan Central Hospital, Cheeloo College of Medicine, Shandong University, Jinan 250013, People’s Republic of China; Department of Oncology, Jinan Central Hospital, Cheeloo College of Medicine, Shandong University, Jinan 250013, People’s Republic of China; Department of Thoracic Surgery, Jinan Central Hospital, Cheeloo College of Medicine, Shandong University, Jinan 250013, People’s Republic of China

**Keywords:** adenocarcinoma of the esophagogastric junction, MMP-2, VEGF, immunohistochemistry

## Abstract

Adenocarcinoma of the esophagogastric junction (AEG) has been increased in recent years and has become a worldwide problem that seriously affects human health. The purpose of the study is to investigate the clinical and prognostic characteristics of the matrix metalloproteinase-2 (MMP-2) and vascular endothelial growth factor (VEGF) expression in AEG patients. A total of 69 patients were enrolled in this study. The result showed that the high expression of MMP-2 was significantly associated with tumor differentiation (*P* < 0.05) and depth of invasion (pT, *P* < 0.05). The high expression of VEGF was significantly associated with pT (*P* < 0.05) and lymph node metastasis (pN, *P* < 0.05). There was a positive correlation between MMP-2 and VEGF expression (*P* < 0.01). The 5-year survival rate for the 69 AEG patients was 40.6% and it was significantly associated with tumor differentiation (*P* < 0.05), pN (*P* < 0.01), pTNM stage (*P* < 0.01), MMP-2 expression (*P* < 0.05), and VEGF expression (*P* < 0.05). Cox multivariate regression demonstrated that tumor differentiation and pN were independent factors for the 5-year survival rate. Our study showed that MMP-2 and VEGF could work synergistically in AEG development.

## 1 Introduction

Adenocarcinoma of the esophagogastric junction (AEG) incidents have increased in recent years and become a worldwide problem that seriously affects human health [1,2]. AEG is defined as the malignant tumor whose center is within 5 cm of the proximal and distal ends of the esophagogastric junction (EGJ). It includes proximal gastric cancer, cardiac cancer, and distal esophageal adenocarcinoma [3]. Due to its particular anatomical location, the pathological type of AEG is adenocarcinoma, while the epidemiological characteristics and clinical symptoms of AEG are consistent with esophageal squamous cell carcinoma [4,5]. At present, more and more scholars regard AEG as an independent malignant tumor that is different from gastric cancer and esophageal adenocarcinoma [6,7]. Because of the high recurrences rates, the prognosis of AEG patients remains poor even after the curative surgery treatment [8,9]. The TNM staging system lacks sufficient implied rate, because significantly different survival rate is often observed in the same TNM stage. Therefore, it is meaningful to combine some biomarkers with TNM staging to distinguish AEG patients with poor prognosis.

Matrix metalloproteinases (MMPs), a family of extracellular zinc-dependent endoproteinases [10], play pivotal roles in tumor infiltration, invasion, and angiogenesis [11,12]. Among the MMPs, MMP-2 acts as a key enzyme that could be related to tumor metastasis and physiologic functions [13]. Vascular endothelial growth factor (VEGF) is an angiogenetic factor produced by cancer cells and could stimulate the growth of endothelial cells [12]. It could promote endothelial cells proliferation and migration, enhance the permeability of blood vessels, promote stromal proteolysis, and reduce endothelial cell apoptosis [13]. It has been reported that VEGF could induce multiple proteases expression including MMPs which leads to extracellular matrixaround vessels [14]. The purpose of the study is to investigate the clinic and prognostic characteristics of MMP-2 and VEGF expression in postoperative AEG patients using both univariate and multivariate analysis, and the MMP-2 and VEGF expression was detected using immunohistochemistry (IHC) at protein level.

## 2 Materials and methods

### 2.1 Patients

Total of 69 AEG cases were enrolled into the study conducted at the Department of Thoracic Surgery and General Surgery, Jinan Central Hospital between January 2010 and May 2013. The inclusion criteria were as follows: (1) patients underwent surgery and affirmed AEG by pathology; (2) the TNM staging system of AEG was based on the International Union Against Cancer (2009) guideline; (3) the subjects had no preoperative chemotherapy or radiotherapy treatment; (4) the cases had no seriously surgical contraindications that could affect prognosis. (5) The follow-up data of the cases were complete. The clinic data of the AEG patients were shown in Table 1. This research was approved by the Ethics Committee of Jinan Central Hospital and was in accordance with the ethical standards of the Helsinki Declaration of 1975, as revised in 2000. And all the patients consented to the study.

**Table 1:** Correlation between MMP-2 and VEGF expression and clinicopathological features of the patients with adenocarcinoma of the esophagogastric junction

Clinical features	Patients	MMP-2			VEGF		
		Low	High	*P*	Low	High	*P*
	69	21	48		20	49	
**Gender**				*0.561			*0.554
Male	50	14	36		16	34	
Female	19	7	12		4	15	
**Age, year**				*0.788			0.234
<60	26	7	19		10	24	
≥60	43	14	29		17	22	
**Differentiation**				*0.036			*0.430
Well + Moderately	32	14	18		11	21	
Poorly	37	7	30		9	28	
**pT**				*0.019			*0.036
pT1 + pT2	34	15	19		14	20	
pT3 + pT4	35	6	29		6	29	
**pN**				*0.286			*0.003
−	28	11	17		14	14	
+	41	10	31		6	35	
**pTNM**				*0.187			*0.106
pI + pII	40	15	25		15	25	
pIII	29	6	23		5	24	
**VEGF**				**0.004			
Low	20	11	9		—	—	
High	49	10	39		—	—	

*P*: *χ* ^2^ test, ^*^Fisher’s exact probability test, ^**^Spearman’s rank correlation coefficient; pT, tumor invasion; pN, lymph node metastasis, pTNM, tumor stage; MMP-2, matrix metalloproteinases 2; VEGF, vascular endothelial growth factor.

### 2.2 Immunohistochemistry

All the AEG specimens were obtained from the 69 cases. The tissue specimens were fixed in 10% neutral buffered formalin and processed routinely. MMP-2 and VEGF were detected by the streptavidin-peroxidase (SP) method using the same paraffin-embedded tissue samples, which were cut into 4-mm-thick slices. The primary antibody was applied using mouse antihuman monoclonal MMP-2 antibodies (1:150, Catalogue TA806846, Zhongshan Jinqiao Biotechnology, Beijing, P. R. China) or mouse antihuman monoclonal VEGF antibodies (1:100, Catalogue ZM-0265, Zhongshan Jinqiao Biotechnology, Beijing, P. R. China). The IHC protocols were described previously [15,16,17].

### 2.3 Immunohistochemical findings evaluation

**Figure 1:**
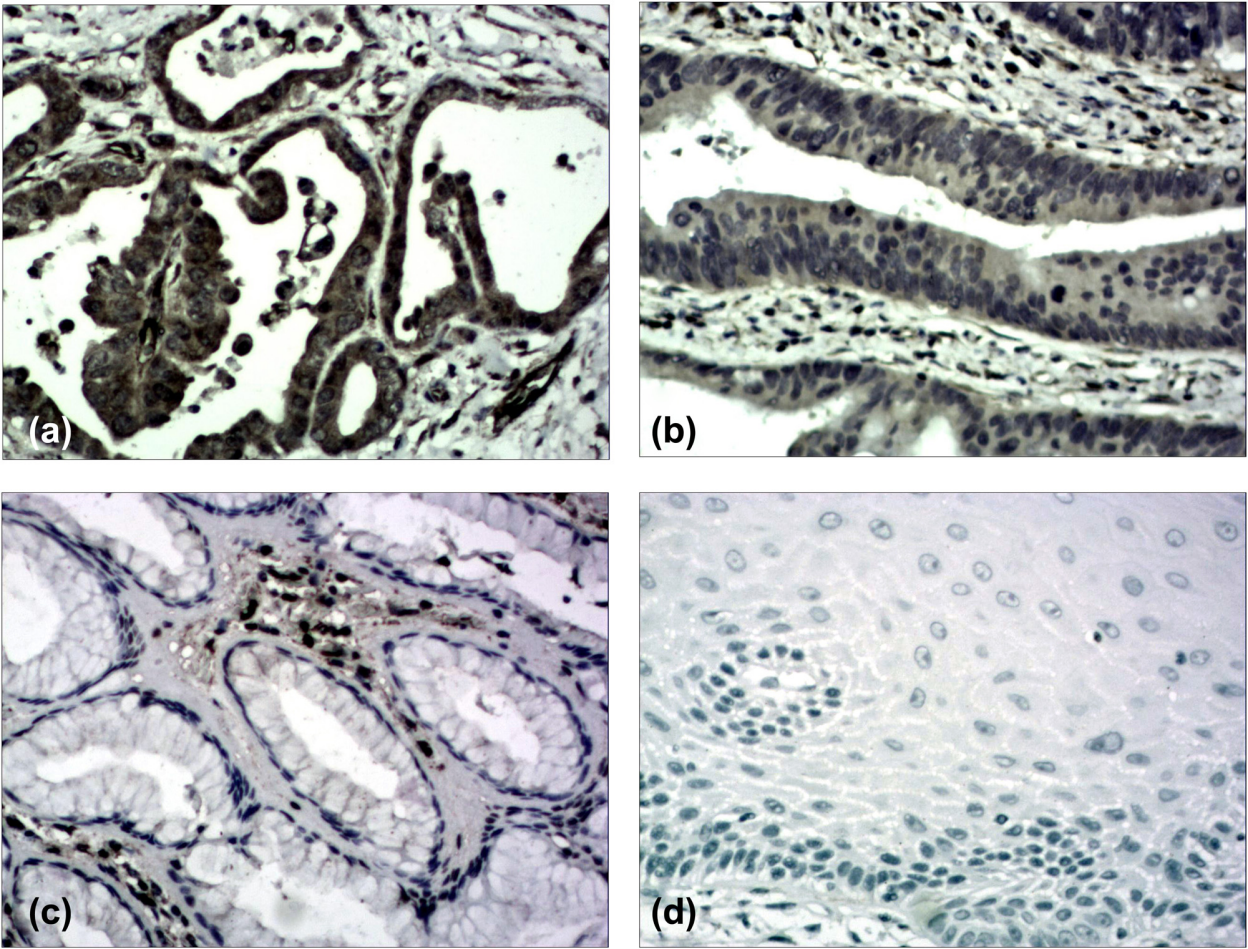
Immunohistochemical staining of adenocarcinoma of the esophagogastric junction (AEG) tissue sections demonstrating Matrix metalloproteinase-2 (MMP-2) (Original magnification ×200). (a) AEG specimen with high expression of MMP-2. (b) AEG specimen with low expression of MMP-2. (c) The corresponding normal gastric tissue specimen with no MMP-2 expression (contrast). (d) The corresponding normal esophageal tissue specimen with no MMP-2 expression (contrast).

**Figure 2:**
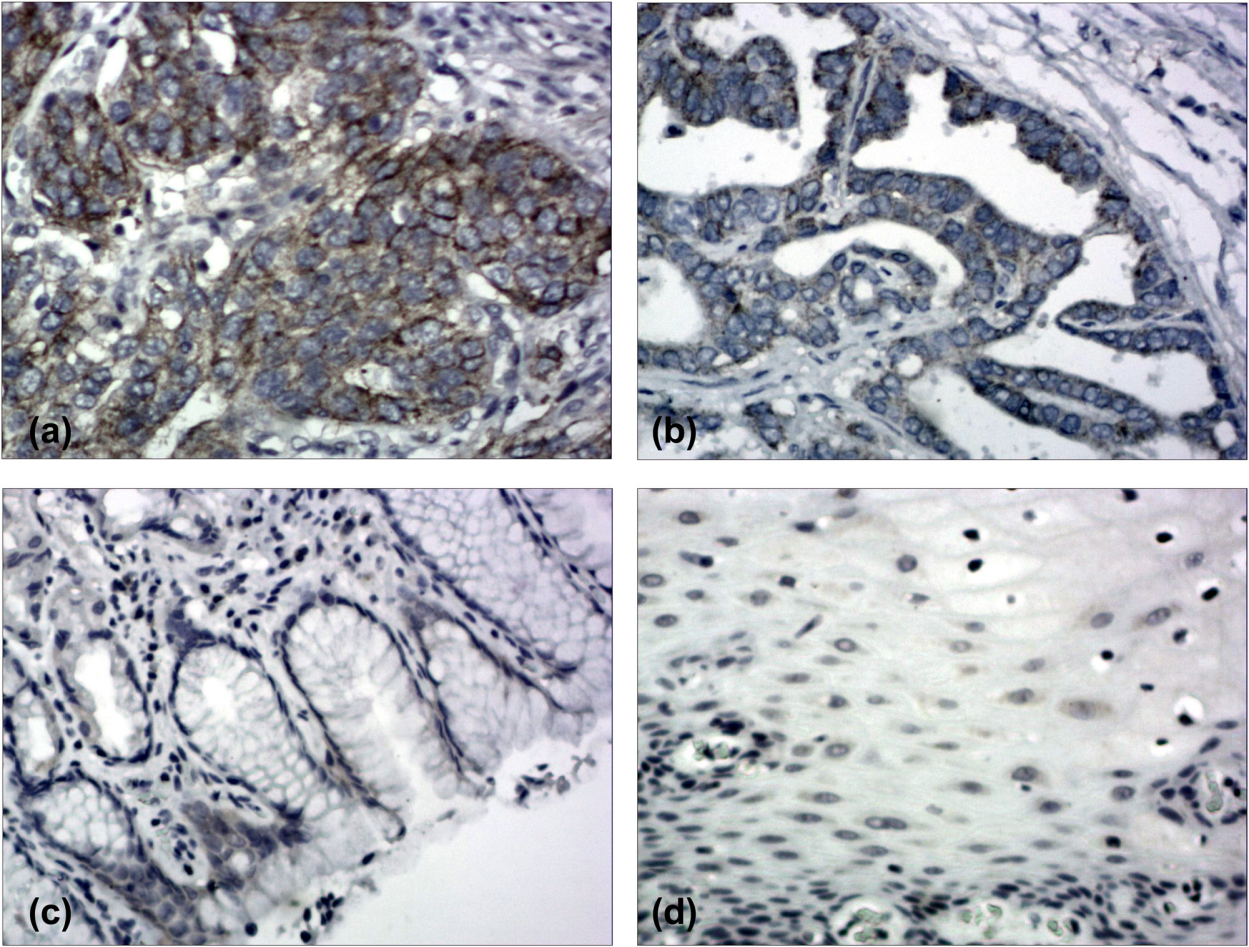
Immunohistochemical staining of adenocarcinoma of the esophagogastric junction (AEG) tissue sections demonstrating vascular endothelial growth factor (VEGF) (Original magnification ×200). (a) AEG specimen with high expression of VEGF. (b) AEG specimen with low expression of VEGF. (c) The corresponding normal gastric tissue specimen with no VEGF expression (contrast). (d) The corresponding normal esophageal tissue specimen with no VEGF expression (contrast).

Cell counts were performed by counting 200 cells in each area of at least 5 randomly selected areas with 400× magnification using a light microscope. MMP-2 expression was mainly located in the cytoplasm and plasma membrane of the tumor cells and categorized under the following conditions: low expression, less than 50% of cells; high expression, 50% or more of cells (Figure 1) [16]. VEGF expression was mainly located in the cytoplasm of the tumor cells and was categorized as follows: staining the rate of tumor cells was scored between 0 and 4: 0 (below 5%), 1 (6–25%), 2 (26–50%), 3 (51–75%), and 4 (above 75%); staining intensity was scored between 0 and 3: 0 (negative), 1 (mildly positive), 2 (moderately positive), and 3 (strongly positive). The final score was obtained by multiplying diffusion and intensity scores. Those with final scores ≤4 were classified as low expression group and those with ≥5 as high expression group (Figure 2) [17].

### 2.4 Statistical analysis

Enumeration data were performed by *χ* ^2^ test or Fisher’s exact probability test. The correlation between MMP-2 and VEGF expression was analyzed using Spearman’s rank correlation coefficient. Univariate analysis was performed with Kaplan–Meier survival curves. Multivariate analysis was performed by the Cox proportional hazard model. All statistical data were analyzed using SPSS (IBM SPSS, Statistics 25, USA), and *P* < 0.05 indicated a statistically significant difference.

### 2.5 Follow-up

Overall, 32 cases had postsurgical chemotherapy, 3 cases had postsurgical radiotherapy, and 19 cases had postsurgical radiotherapy combined with chemotherapy. Patients who died of tumor were included in the prognostic analysis.

## 3 Results

The high expression of MMP-2 was significantly associated with tumor differentiation (Well + Moderately 56.3% vs Poorly 81.1%; *P* < 0.05) and depth of invasion (pT; pT1 + pT2 55.9% vs pT3 + pT4 82.9%; *P* < 0.05). No statistically significant correlations with gender, age, pN, and pTNM stage were demonstrated for MMP-2 (*P* > 0.05). The high expression of VEGF was significantly associated with pT (pT1 + pT2 58.9% vs pT3 + pT4 82.9%; *P* < 0.05) and pN (pN – 50.0% vs pN + 85.4%; *P* < 0.05). No statistically significant correlations with gender, age, tumor differentiation, and pTNM stage were demonstrated for VEGF (*P* > 0.05). There was a positive correlation between MMP-2 and VEGF expression (*P* < 0.01) (Table 1).

**Figure 3:**
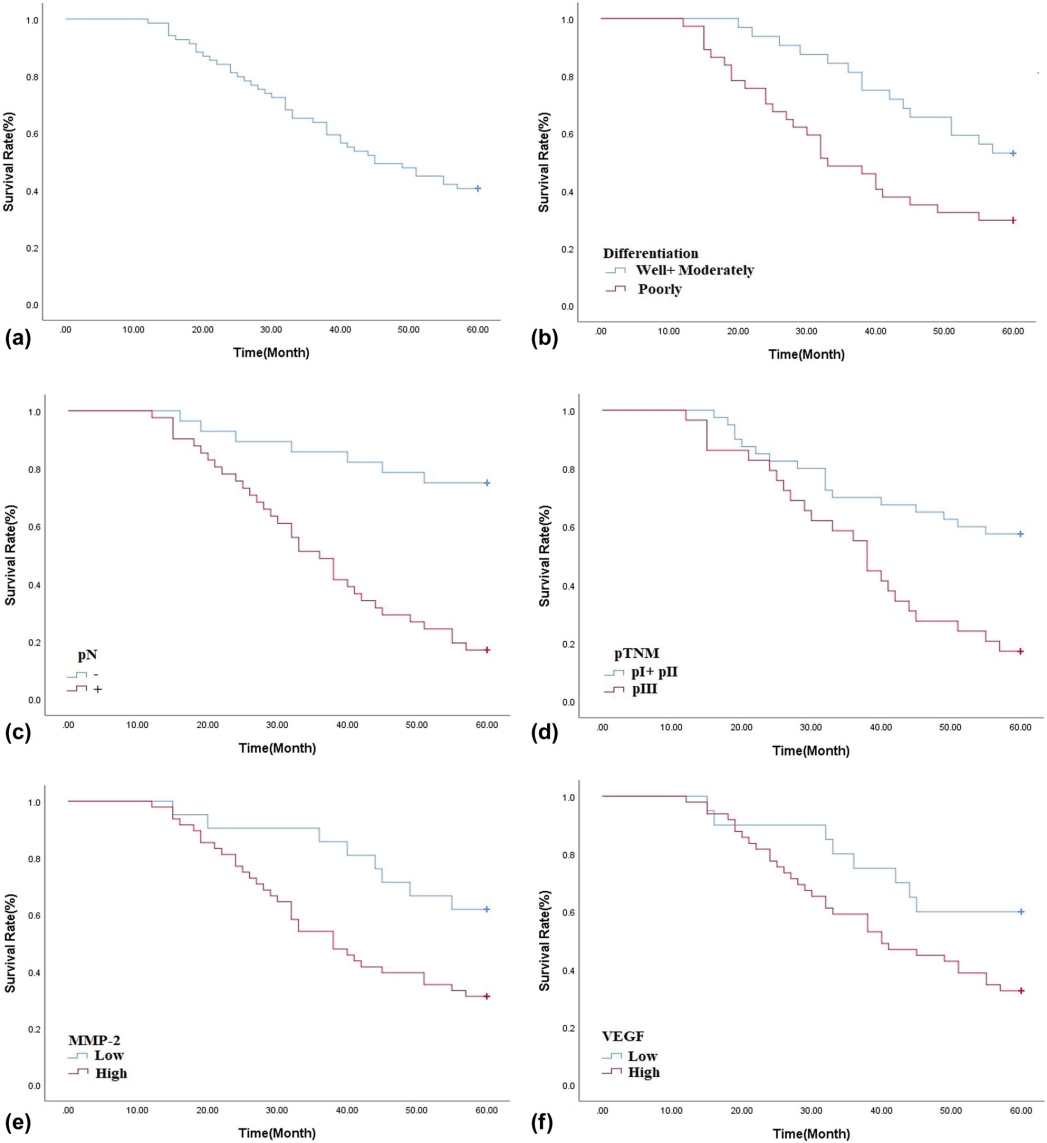
(a) The Kaplan-Meier survival curve of 69 cases of AEG patients. (b) Survival curves of AEG patients with tumor differentiation. (c) Survival curves of AEG patients with negative or positive pN. (d) Survival curves of AEG patients with different pTNM. (e) Survival curves of AEG patients with low or high expression of MMP-2 expression. (f) Survival curves of AEG patients with low or high expression of VEGF expression.

The 5-year survival rate for all the 69 AEG patients was 40.6%. A univariate analysis was conducted using the log-rank test, and the 5-year survival rate was significantly associated with differentiation (*P* < 0.05), pN (*P* < 0.01), pTNM stage (*P* < 0.01), MMP-2 expression (*P* < 0.05), and VEGF expression (*P* < 0.05) (Figure 3, Table 2). The 5-year survival rate of patients with low MMP-2 expression in AEG tissues was significantly higher than those with high MMP-2 expression (61.9% vs 31.3%; *P* = 0.013). Similarly, the 5-year survival rate of patients with low VEGF expression in AEG tissues was significantly higher than those with high VEGF expression (60.0% vs 32.7%; *P* = 0.048). The Cox multivariate regression result showed that both tumor differentiation and pN were independent factors for the 5-year survival rate (Table 3).

**Table 2:** Univariate analysis with respect to 5-year survival of the patients with adenocarcinoma of the esophagogastric junction

Clinical features	Patients	5-year survival (%)		
		Patients	Rate (%)	*P* value
	69	28	40	6
**Gender**				0.617
Male	50	20	40.0	
Female	19	8	42.1	
**Age, year**				0.223
<60	26	8	30.8	
≥60	43	20	46.5	
**Differentiation**				0.011
Well + moderately	32	17	53.1	
Poorly	37	11	29.7	
**pT**				0.183
pT1 + pT2	34	16	47.1	
pT3	35	12	34.3	
**pN**				0.001
−	28	21	75.0	
+	41	7	17.1	
**pTNM**				0.001
pI + pII	40	23	57.5	
pIII	29	5	17.2	
**Chemotherapy**				0.946
No	18	8	44.4	
Yes	51	20	39.2	
**Radiotherapy**				0.896
No	47	19	40.4	
Yes	22	9	40.9	
**MMP-2**				0.013
Low	21	13	61.9	
High	48	15	31.3	
**VEGF**				0.048
Low	20	12	60.0	
High	49	16	32.7	

*P*, log-rank test; SCC, squamous cell carcinoma; ADC, adenocarcinoma; pT, tumor invasion; pN, lymph node metastasis, pTNM, tumor stage; MMP-2, matrix metalloproteinases 2; VEGF, vascular endothelial growth factor.

**Table 3:** Results of Cox regression multivariate 5-year survival analysis of the patients with adenocarcinoma of the esophagogastric junction

	*B*	SE	Wald	*P*	HR	95.0% CI for HR
Gender	−0.554	0.384	2.083	0.149	0.575	0.271–1.219
Age	0.104	0.356	0.086	0.769	1.110	0.553–2.229
Differentiation	0.885	0.364	5.911	0.015	2.424	1.187–4.950
pT	0.803	0.437	3.370	0.066	2.232	0.947–5.259
pN	2.494	0.632	15.545	0.001	12.104	3.504–41.809
pTNM	−0.671	0.487	1.899	0.168	0.511	0.1197–1.328
Chemotherapy	−0.245	0.429	0.325	0.568	0.783	0.338–1.815
Radiotherapy	0.050	0.369	0.019	0.891	1.052	0.510–2.168
MMP-2	0.448	0.436	1.053	0.305	1.565	0.665–3.681
VEGF	−0.441	0.471	0.876	0.349	0.644	0.256–1.619

*B*, regression coefficient; SE, standard error; Wald, Wald value; HR, hazard ratio; CI, confidence interval; pT, tumor invasion; pN, lymph node metastasis, pTNM, tumor stage; MMP-2, matrix metalloproteinases 2; VEGF, vascular endothelial growth factor.

## 4 Discussion

Matrix metalloproteinase 2 (MMP-2), as one of the MMPs family members, has been confirmed to promote invasion and metastasis in many kinds of tumors [18,19]. Reports show that MMP-2 is highly expressed in cancers such as ovarian cancer [20], renal cell carcinoma [21], and prostate cancer [22], leading to poor survival of patients. However, the prognostic characteristics of MMP-2 expression in tumor patients remain controversial. In Pellikainen JM’s report, the high MMP-2 expression in cancer cells had no prognostic value for breast cancer patients [23]. By contrast, Shen et al. [24] had the opposite conclusion. They found that MMP-2 overexpression was a predictive factor for poor prognosis of gastric cancer. Qian et al. [25] and Liu et al. made the [26] same conclusions as Shen’s by studying non-small cell lung cancer (NSCLC) and endometrial cancer, respectively. However, little research has been done for the MMP-2 clinical features in AEG patients. Lu et al. [27] detected MMP-2 expression using IHC in tumors specimens from 120 AEG patients. They found that 51.7% of the cases had MMP-2 overexpression. However, no significant associations were found between MMP-2 and clinicopathological features in these AEG patients. Based on the study of 69 AEG patients, our results showed that the high expression of MMP-2 was significantly associated with tumor differentiation and depth of invasion (pT). The 5-year survival rate of AEG patients was 40.5%, and it was significantly associated with MMP-2 expression by the univariate analysis. However, MMP-2 expression was not the relevant independent factor for a poor prognosis in multivariate analysis. Our conclusion was different from Liu X’s. The reason could be attributed to many factors which could affect the experimental results. For example, the techniques used to detect MMP-2 expression might be one important reason for potential bias. Although IHC was the most frequent technique used in clinical research, the results of IHC depend on the primary antibody. Differences in antibody, antibody dilution, and cut-off of defining the specimens as MMP-2 positivity could result in potential heterogeneity. Unfortunately, up to now, there was no common threshold in defining MMP-2 positive expression in AEG patients. It is very important to set a standard threshold in assessment of biomarkers, such as MMP-2, to make the best evaluation of their real function in clinical practices. In order to become a useful prognostic factor at the level of individual patients, our conclusion needs to be further confirmed by an adequately designed prospective research. Moreover, the exact MMP-2 expression value should be determined by both univariate and multivariate analysis in light of the well-established prognostic factors for AEG.

VEGF is an angiogenetic factor, which could promote endothelial cells proliferation and migration, enhance blood vessels permeability, and reduce apoptosis of endothelial cells [28,29]. VEGF is correlated with invasion and metastasis in many kinds of cancers, including esophageal and gastric cancer [30,31]. However, few studies have explored the clinical features of VEGF in AEG patients. Gray et al. [32] detected VEGF expression using IHC in tumors specimens from 61 esophagogastric patients. They found that VEGF was overexpressed in tumor epithelial cells. However, it had no prognostic value for esophagogastric cancer patients. Park et al. [33] also detected VEGF expression in serum levels of ligands from 147 patients who underwent potentially curative resection for gastric and esophagogastric adenocarcinoma. They found that VEGF levels were higher in patients with R1 vs R0 resections. The increased VEGF levels were correlated with decreased overall survival rate. Moreover, the serum VEGF was found as a significantly independent prognostic factor for overall survival. The controversy of the above findings could be possibly due to the different tissue specimens used and different stage or analytic method employed. Even using the same analytic method, the result may differ depending on the site selected for assessment. In our study, the VEGF protein expression in all the patients was detected by IHC. Our data showed that the high expression of VEGF was associated with both tumor depth of invasion (pT) and lymph node metastasis (pN). The 5-year survival rate of AEG patients was associated with VEGF expression by univariate analysis. To eliminate the impact of mixed factors on statistical analysis, we used multivariate analysis to determine prognostic factors, and our result showed that tumor differentiation and pN were relevant independent factors for a poor prognosis.

Recently, some reports showed there was a relationship between VEGF and MMPs in tumor progression. Zhang et al. [34]found that in vitro induction and activity of MMP-2 stimulated by VEGF might be the main mechanism by which VEGF gave impetus to ovarian cancer cells invasion. Wang et al. [35] confirmed that anti-basic fibroblast growth factor (anti-bFGF)-induced invasion of human lung cancer cells could be rescued by inhibiting the AKT/MMP-2/VEGF loop. Partyka et al. [36] confirmed that there was a significantly positive correlation between VEGF and MMP-2 in gastric cancer tissue of patients with metastases. So far, the correlation between VEGF and MMPs in AEG has not been reported in PubMed. In our study, 69.6% AEG cases had high MMP-2 expressions, 71.0% AEG with high VEGF expression, and 56.5% AEG cases had both high expressions of VEGF and MMP-2. Our study showed that MMP-2 expression was positively related to VEGF expression in AEG tissues. We concluded that MMP-2 and VEGF could work synergistically in AEG development.

This is the first report to study the relationship between VEGF and MMP-2 in AEG at clinical level. In our study, all the patients successfully underwent radical operation with regional lymph node dissection. The tumor did not invade other organs, and both edges of resection were confirmed to be free of residual cancer cells by routine histological examination, to ensure complete resection. To eliminate the impact of mixed factors correlated with prognosis on statistical analysis, the Cox regression multivariate analysis was performed to determine the independent prognostic factors. As a result, the comparability was increased and statistical bias was decreased, making the results of this study more objective.

However, the present study still has several limitations. First, in China the indications for treatment not only depend on doctors’ preferences, but also patients’ willingness and economic status. These factors may have influenced the relatively poor survival result observed. In the study, 32 patients received postoperative chemotherapy, 3 patients received postoperative radiotherapy, and 19 patients received combined chemoradiotherapy. However, no statistically significant correlations with postoperative chemotherapy and radiotherapy were demonstrated for the 5-year survival rate either in univariate or multivariate analysis. Second, this is a retrospective study with a small sample size, which could limit the value of the findings. A randomized-controlled prospective study with a larger sample size will be considered in further investigations.

In conclusion, high MMP-2 expression was significantly associated with tumor differentiation and depth of invasion in AEG patients. In addition, the high expression of VEGF was significantly associated with tumor depth of invasion and lymph node metastasis. There was a positive correlation between MMP-2 and VEGF expressions. Collectively, the results suggest that MMP-2 and VEGF could work synergistically in AEG development.
